# Brain Involvement in Leishmaniasis

**DOI:** 10.1002/cbf.70209

**Published:** 2026-04-11

**Authors:** Camila S. Freitas, Eduardo A. F. Coelho, Myron Christodoulides

**Affiliations:** ^1^ Programa de Pós‐Graduação em Ciências da Saúde: Infectologia e Medicina Tropical, Faculdade de Medicina Universidade Federal de Minas Gerais, Belo Horizonte Minas Gerais Brazil; ^2^ Departamento de Patologia Clínica, COLTEC Universidade Federal de Minas Gerais, Belo Horizonte Minas Gerais Brazil; ^3^ Molecular Microbiology, School of Clinical and Experimental Sciences University of Southampton Faculty of Medicine, Southampton General Hospital Southampton England

**Keywords:** blood‐brain barrier, blood‐cerebrospinal fluid barrier, brain, canine, cutaneous, human, inflammation, Leishmania, leishmaniasis, visceral

## Abstract

Leishmaniasis is a neglected tropical disease caused by infection with the protozoan parasite *Leishmania* and it is a significant global health problem. The disease has a wide clinical spectrum, from tegumentary leishmaniasis (TL) that encompasses cutaneous (CL), mucosal (ML) and cutaneous‐diffuse (CDL) forms, to the potentially fatal systemic visceral leishmaniasis (VL). Neurological manifestations are not generally considered as classical clinical signs or symptoms of leishmaniasis, but in this review, we present evidence that this is a false assumption. We examine brain involvement in human and canine leishmaniasis and the contribution of animal models for studying cerebral *Leishmania* infection. Clinical descriptions of brain involvement are presented, and evidence of *Leishmania* invasion of the central nervous system. Notably, evidence for brain involvement comes from considerable studies in the dog and covers aspects of disruption of the blood‐brain and blood‐cerebrospinal fluid barriers and the nature of the inflammatory response.

## Summary

Leishmaniasis is a disease endemic across several continents, with an estimated 1.5 to 2.0 million new cases and 70,000 deaths annually. The disease has a wide clinical spectrum, ranging from cutaneous or mucosal lesions characterised as tegumentary leishmaniasis (TL), to potentially fatal systemic visceral leishmaniasis (VL). There is substantial evidence that *Leishmania* infection can effect changes in the central and peripheral nervous systems, and neurological manifestations should not be dismissed as potential supporting diagnostic markers.

## 1 Introduction

Leishmaniasis is a vector‐borne disease caused by protozoan parasites of the genus *Leishmania*, which are transmitted by the bite of infected female phlebotomine sand flies. It is an important neglected tropical disease (NTD) with a significant global health burden [1]. The disease is endemic in over 90 countries across Asia, Africa, the Americas, the Mediterranean, and parts of Southern Europe, with an estimated 1.5 to 2.0 million new cases and 70,000 deaths annually [2, 3]. Leishmaniasis affects mammalians such as canids and humans, with dogs representing the main domestic reservoirs for zoonotic visceral leishmaniasis (VL). The disease presents a wide clinical spectrum, ranging from cutaneous or mucosal lesions termed as tegumentary leishmaniasis (TL), to the potentially fatal systemic VL [1].

TL presents in different clinical forms: (1) the localised cutaneous form (CL) is characterised by one or more ulcerated skin lesions at the site of the sandfly bite; (2) the mucosal form (ML) involves destructive lesions of the mucous membranes, particularly in the nose, mouth, and throat; and (3) the cutaneous‐diffuse form (CDL) is marked by multiple non‐ulcerative nodules spread across the body, often associated with a poor immune response to the parasite [4].

More than 20 *Leishmania* species are pathogenic to mammals, and they are grouped according to the clinical syndromes they cause. VL is caused by *L. donovani* and *L. infantum* species, CL is caused by diverse species such as *L. major*, *L. tropica*, *L. mexicana* complex, *L. braziliensis* complex, amongst others, and ML is primarily associated with the *L. braziliensis* complex [4]. Dogs are susceptible to *L. infantum* infection and play a critical role in maintaining the zoonotic transmission cycle [5]. The clinical manifestations depend mainly on the *Leishmania* species involved and the host immune response [6] and are summarised in Table 1.

**Table 1:** Main clinical signs of leishmaniasis in humans and dogs.

Host	Clinicalform	Main clinical signs/symptoms
Human	Visceral (VL)	Prolonged fever, weight loss, appetite loss, enlargement of the spleen and liver (splenomegaly, hepatomegaly), anaemia, and can be fatal if untreated [7, 8].
	Cutaneous(CL)	Characterised by localised skin lesions, papules, nodules, or ulcers at the site of the sand fly bite. Lesions may be painless or painful and can become chronic or disfiguring [1].
	Mucosal (ML)	Involves destruction of mucous membranes of the nose, mouth and throat, leading to severe facial disfigurement and difficulty in eating or breathing [8].
Dog	Canine leishmaniasis	Skin lesions (alopecia, ulcers), lymphadenopathy, weight loss, onychogryphosis, ocular involvement, epistaxis, renal impairment [9].

**Figure 1:**
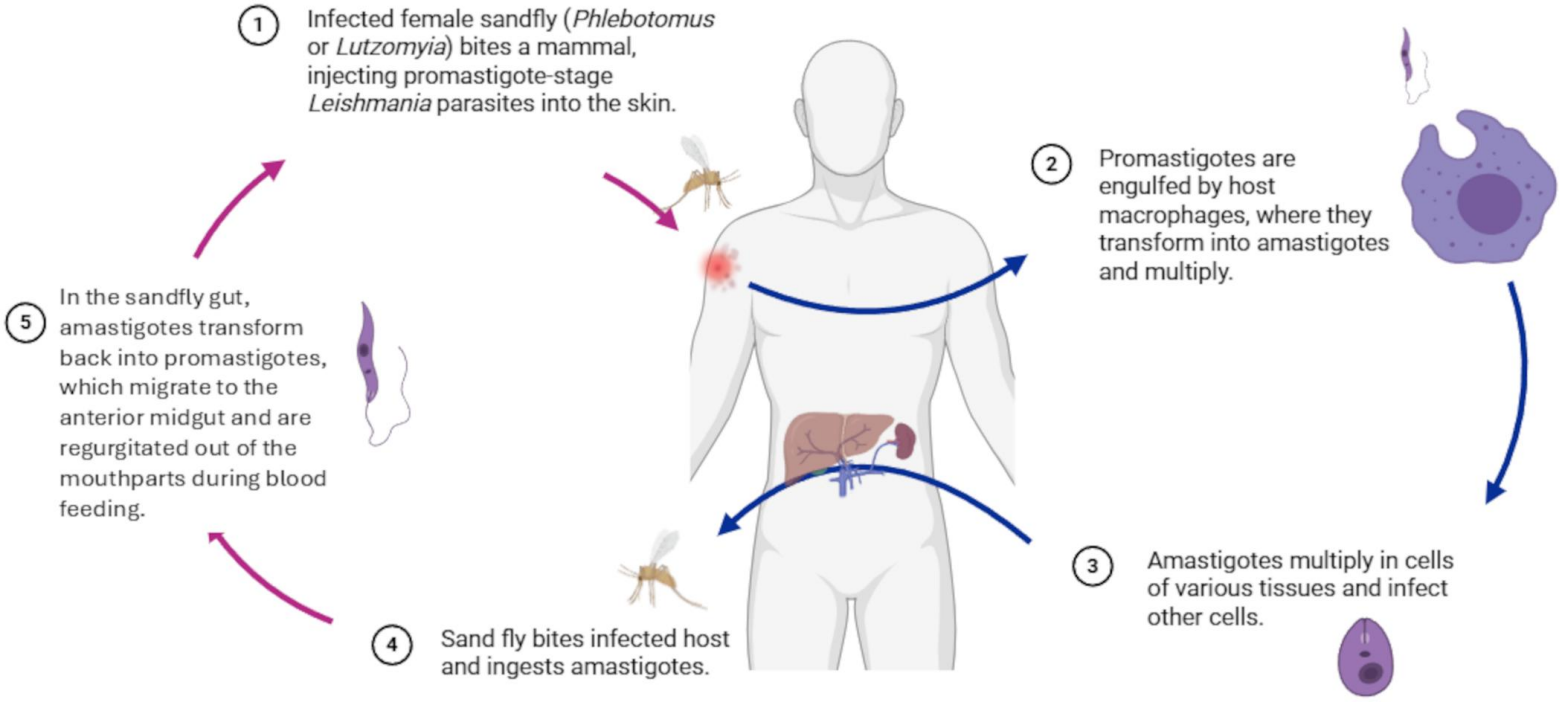
Life cycle of the *Leishmania* parasite in mammals. Leishmaniasis is a zoonotic infection, and the dog acts as a reservoir for the parasite *L. infantum*. Transmission between mammals of promastigotes occurs through the bite of the infected female sandfly. Promastigotes are engulfed by macrophages and therein they transform into amastigotes. Amastigotes also infect other cell types and tissues. Transmission continues when sandflies bite their mammalian hosts again and take up amastigotes, which migrate mainly to the anterior midgut, revert to promastigotes, and the cycle continues when they are regurgitated out of the mouthparts during blood feeding.

The pathogenesis of leishmaniasis is complex and involves both parasite infectivity and host immune responses, and a full description is outside the scope of this review. Briefly, the life cycle begins when a sandfly inoculates promastigotes into the skin during a blood meal. Once inside the host, *Leishmania* promastigotes are engulfed by phagocytic cells (mainly neutrophils and macrophages), where they transform into *Leishmania* amastigotes that multiply and disseminate to various tissues (Figure 1) [6]. The disease progression depends on the balance of the host's immune response. A Th1‐type cellular response, characterised by the production of IFN‐γ, IL‐2 and TNF‐α cytokines, amongst others, is associated with parasite clearance and resistance against infection. Conversely, a Th2‐type cellular response, based on the production of IL‐4, IL‐10 and TGF‐β cytokines, amongst others, favours parasite persistence and disease progression [10, 11, 12, 13]. Cytokines such as IL‐27 and IL‐1β play dual roles, either protecting against disease or exacerbating disease, depending on host immunity and the parasite species [14]. In addition, an excessive or dysregulated immune response can also cause tissue damage and immunopathology [13, 15].

To favour their survival, *Leishmania* have evolved strategies to evade or manipulate host immune defenses, including modulating host transcription factors (e.g., YY1, Argonaute 1) and altering host gene expression [16, 17, 18]. The parasites can also hijack myeloid cells and delay or suppress effective immune responses [19, 20]. Virulence factors include surface proteins and secreted molecules, which enable immune evasion and modulation of host cell functions [16, 20]. Additionally, sand fly gut microbes introduced during the bite can enhance disease severity by promoting inflammatory responses that benefit the parasite [21]. In humans, the immune balance determines whether infection results in self‐healing cutaneous lesions, disfiguring mucosal disease or life‐threatening visceral infection. In dogs, the disease is often chronic and systemic, with renal pathology being the major cause of death [5, 22].

Neurological manifestations are not generally considered as classical clinical signs or symptoms of leishmaniasis and consequently, involvement of the brain is rare and rarely reported. Our contention is that this assumption is false, and neurological manifestations are probably under‐reported, with compelling evidence available for both humans and dogs. In this review, we examine brain involvement in human and canine leishmaniasis and the contribution of animal models for studying cerebral parasite infection.

## 2 Brain Involvement in Human Leishmaniasis

In agreement with Llanos‐Cuentas et al. [23] and Petersen and Greenlee [24], neurological involvement in human cases of leishmaniasis can be classified into four groups: (1) peripheral demyelinating immune‐mediated neuropathy in VL, (2) peripheral neuropathy in CL patients by direct or close parasite involvement with the nerve or nerve sheath, (3) central nervous system (CNS) involvement by hematogenous dissemination in VL or close proximity in ML, and (4) neuropathy associated with anti‐*Leishmania* treatments. A summary of historical and recent case reports for human VL with neurological manifestations is presented in Table 2. The clear signs of mental disturbances, sensory impairment, depression, apathy, and peripheral nervous effects on the lower limbs, which are seen in patients with leishmaniasis (Table 2), are strongly indicative of parasite perturbation of normal CNS and peripheral nervous system (PNS) functions.

**Table 2:** Case reports of neurological manifestations in humans with leishmaniasis.

Classification	Case	Clinical and histopathological findings	Citation
1. Peripheral demyelinating immune‐mediated neuropathy in VL	VL patients13 cases in Khartoum, Sudan	Neurological abnormalities confined to lower limbs: pain in the soles, burning, itching, throbbing; footdrop; neuropathy; mental disturbances (visual hallucinations and delusions); sensory impairment and vibration loss; mild plantar hyperalgesia. One patient with spinal cord involvement.	[25]
	111 patients with VL, Khartoum, Sudan.	52 patients had neurological symptoms or signs, commonly a sensation of burning feet; 4 patients had foot drop; 5 patients had deafness; 1 patient had multiple cranial nerves palsies. Evidence of axonal degeneration and demyelination in 15 patients. But no direct parasitic infection of nerves or neuritis. Anti‐leishmanial treatment abrogated these symptoms.	[26]
2. Peripheral neuropathy in CL by direct or close parasite involvement with the nerve or nerve sheath	Review of CL lesions caused by *L. major*	Amongst 288 skin biopsy specimens from CL lesions, 14 showed assorted nerve changes; 10 patients with perineural inflammatory cell infiltrate (lymphocytes or lymphocytes/plasma cells/macrophages mixture); 4 patients had neuritis; one patient had granulomatous neuritis and nerve destruction. Amastigotes observed inside the nerves in two patients; 2 patients had diminished sensations over the CL lesions.	[27]
	Male presenting with CL‐characteristic tender hyperaesthetic skin nodules.	Lymphohistocytic inflammatory infiltrate observed around and within cutaneous nerves and presence of *Leishmania* parasites in the perineural space.	[28]
	45‐year‐old male with ML/HIV	Optic neuropathy and sinus lesion extending into orbital apex; intracellular parasites in nasal and sphenoidal mucosa.	[29]
	Brazilian patient with CL	Presence of parasites in anterior eye chamber. Ocular involvement was a bilateral nongranulomatous iridocyclitis.	[30]
3. CNS involvement by hematogenous dissemination in VL or close contiguity in ML	VL patients in India, East Africa, Northern Africa	Amastigotes present in meninges, endothelia of meningeal vessels and in the CSF. Associated paralytic symptoms. Mental changes not unusual	[31]
	VL patients (223 cases) in Kitui District, Kenya	Curious (*sic*) mental depression and complete apathy.	[32]
	VL patients in Ethiopia	3/18 patients had ‘definite mental changes’	[33]
	10‐yr old boy in Bihar, India	Presented with symptoms of meningitis; CSF contained leishmanial amastigotes, and patient was finally cured with a course of amphotericin B, which can cross the BCSFB	[34]
	VL associated with AIDS in a 32‐year‐old man, Brazil	Brain had oedema, congestion in leptomeninges, microglial proliferation, but no parasites detected. Antigenic material related to *Leishmania* was positive in brain samples (microglial cells containing amastigotes).	[35]
	HIV/VL infected Algerian mother (40 years) and congenital VL in newborn infant	MRI of infant at 1.5 months revealed cerebral and cerebellar atrophy. First report of hepatic and neurologic impairment in a newborn with congenital VL.	[36]
	A 54‐yr old man from Southwest of Iran (Yasuj) with VL	Axial fluid‐attenuated inversion recovery MRI image (FLAIR) revealed an increase in signal intensity of central part of right side of brain parenchyma pons. No neurological morbidity and disability after treatment with amphotericin B.	[37]
	Male presenting with VL	Defined as a case of myasthenia gravis, presenting with pancytopenia induced by azathioprine drug treatment, coincident with VL masquerade.	[38]
	10‐month‐old male infant, Oman	Diagnosed with Haemophagocytic Lymphohistiocytosis (HLH) with VL.	[39]
	VL patients in Kenya	3 patients developed bilateral anterior uveitis, giving rise to secondary glaucoma in 2 of the patients. In third patient, eye lesions were associated with an episode of post kala azar dermal leishmaniasis.	[40]
	VL patients in Sudan	Bilateral anterior uveitis in 2 patients after successful treatment of kala azar. Parasites and inflammation observed in the eye.	[41]
	Chinese kala azar patients	6/160 patients showed retinal haemorrhages	[42, 43]
	30‐year‐old male patient with kal azar in Kikiyu, Kenya	Retinal haemorrhages in both eyes	[44]
	39‐year‐old man with VL	Retinal haemorrhages in both eyes	[45]
	Brazilian patient with simultaneous VL/CL/ocular	Kidney transplant patient showing conjunctive hyperaemia, intense ocular pain and low visual acuity. Parasites isolated from iliac crest, aqueous humour and vitreous body.	[46]
	Two Italian renal transplant patients with VL/ML	Endophthalmitis (inflammation of intraocular vitreous and aqueous fluids)	[47]
	CL localised on the left upper eyelid in a 36‐year‐old woman.	Single, asymptomatic nodule, diagnosed as an inflammatory infundibular cyst. Histopathology showed an inflammatory infiltrate of lymphocytes, histiocytes, and plasma cells. *Leishmania* spp amastigotes observed in the cytoplasm of macrophages.	[48]
4. Neuropathy associated with anti‐*Leishmania* treatments.	Reactivation of Varicella zoster in a 20‐year‐old man with VL	Herpes lesions appearing during or after cessation of Sb5+ treatment. Meningeal varicella‐zoster also reported. Viral reactivation possibly associated with reduction in the absolute number of total leucocytes/lymphocytes/lymphocyte subsets (although the percentage of the total lymphocyte population for each lymphocyte subset did not change significantly).	[49, 50, 51, 52]
	38‐year‐old male deployed to Iraq for military operations with CL.	CNS cognitive deficits on treatment with L‐AMB, probably side‐effect of the anti‐leishmanial drug. Mental status returned to baseline after completion of drug therapy.	[53]

The pathogenesis of peripheral demyelinating immune‐mediated neuropathy in VL is unclear, but neurological deficits resolve completely following antileishmanial treatment [26]. However, its consideration as a Guillain‐Barre‐like neuropathy associated with VL remains confusing [54, 55]. In the second group, inflammation of peripheral nerves adjacent to CL lesions, lymphatic involvement adjacent to perineural sheaths and the direct involvement of the parasite help to explain the pathogenesis [27, 28], and pain associated with the lesions is alleviated with antileishmanial treatment. In the third group, CNS involvement is infrequently reported in VL, but it can be further classified to meningo‐encephalic involvement and ocular leishmaniasis [23], with the latter rather common (Table 2). It appears that there are clear biological and clinical links between CNS involvement and ocular involvement: (1) they arise from a shared pathophysiology, in that VL has documented CNS complications and predisposes to ocular disease; (2) ocular involvement may occur via the haematogenous route or local spread, the same mechanisms that underlie CNS invasion; and (3) systemic immune dysregulation in VL contributes to both ocular and CNS inflammation that is driven by host–parasite interactions. Anterior uveitis is the most common ocular manifestation in humans, and it can occur either prior to, or just after successful treatment of VL. Parasites are infrequent within the orbit [40, 41], but a case has been reported of the isolation of a *Leishmania* spp. from the aqueous humour of a patient with CL and bilateral non‐granulomatous iridocyclitis [30]. Uveitis has been reported to progress to secondary glaucoma with increased intraocular pressure and potential optic nerve damage [40], and intraretinal haemorrhages have also been observed [44, 45]. In Table 2, further descriptions are provided of unusual cases of optic neuropathy and sinusitis and cutaneous/ocular manifestations of leishmaniasis following renal transplantation with immunosuppression. Meningoencephalitis and ocular leishmaniasis in VL probably develop following haematological dissemination of the parasite coupled with immunosuppression, and immediacy of the parasite to nervous tissue in ML patients probably explains the observed CNS involvement [23]. Treatment with anti‐*Leishmania* biologicals generally cures patients with CNS involvement [23]. The final group considers neuropathy induced by the drugs used for treating leishmaniasis, e.g. pentavalent antimonials such as sodium stibogluconate (Sb5 + ), aromatic diamidines such as pentamidine, and liposomal amphotericin B (L‐AMB) (Table 2).

However, direct observation of *Leishmania* parasites in human CNS tissues is rare, and few studies have demonstrated this. Parasites have been observed in the parenchyma pons (brainstem) of a 54‐yr old man from Southwest of Iran with VL [37], and amastigotes have also been observed inside nerves [27]. The direct identification of amastigotes within CSF‐filled spaces and the meninges has also been demonstrated [31, 34]. More direct evidence of the presence of *Leishmania* parasites within CNS tissues is available from studies of canine leishmaniasis, described below.

### 2.1 Brain Involvement in Canine Leishmaniasis

In the early literature, the involvement of the brain in canine VL (CVL) was contradictory. Some studies reported the presence of parasites in all parts of the canine body except in the CNS, whereas others described parasite antigens and immunoglobulins deposited in the interstitial and intravascular spaces of the choroid plexus (CP) in dogs with VL [56]. These early studies described the consequences of these deposits, namely choroiditis that was distinguished by hypercellularity, congestion of capillaries with a build‐up of amyloid, the infiltration of inflammatory cells, epithelial metaplasia (i.e. cell replacement) and thrombosis [57, 58]. Other pathological changes included a satellitosis of glial cells around neurons, neuronophagia, degeneration of pyramidal neurons and necrosis and oedema and increased amyloid in the Virchow‐Robbin space [56, 59, 60]. In an early study of an endemic focus of CVL occurring in North Central Oklahoma, no parasites were found in the brain of a 7‐year‐old female American Foxhound on autopsy, but histopathology recorded diffuse nonsuppurative meningoencephalitis with scattered areas of liquefaction necrosis in grey matter, diffuse vacuolation in the white matter and gliosis in both white and grey matter [61].

The evidence for significant neurological changes in CVL now appears to be irrefutable from the clinical presentations and symptoms and the histopathological findings reported in many case studies [62]. For example, a 14‐month‐old female crossbreed dog with VL presented with acute paraplegia and was diagnosed with renal failure with pelvic limb lower motor neuron signs. Histopathology showed leukocytoclastic vasculitis in multiple organs, malacia (abnormal softening) and haemorrhage in the spinal cord associated with multiple foci of vasculitis within nervous tissue, and spinal cord haemorrhage and paralysis caused by rupture and thrombosis of inflamed vessels [63]. In a detailed study of dogs with *L. chagasi* VL, which compared 21 dogs with neurological involvement and 18 without, the animals suffered generalised seizures, eye disturbances (i.e. blindness, anisocoria and bilateral mydriasis, paralysis of the cranial nerves (strabismus, facial ptosis, dysphagia), vestibular and cerebellar involvement (with head tilting, nystagmus, motor non‐coordination, falls and tremors), paraparesis, tetraparesis and tetraplegia, myoclonus, vocalisation, circle walking and tail chasing [56]. Histopathology showed neuronal degeneration with neurophonagia, focal to diffuse gliosis, leptomeningitis, vascular congestion, perivascular lymphoplasmacytic infiltration, focal microhaemorrhages, white matter vacuolisation and the presence of Gitter cells (i.e. phagocytic microglial cells). CVL can cause cerebrovascular alterations, such as vasculitis, that might predispose dogs to brain infarcts, with magnetic resonance imaging (MRI) images of the brains of dogs infected with *Leishmania* spp. showing multifocal, non‐haemorrhagic, ischaemic lesions [64].

In a singular case report, a 4‐year‐old male Labrador Retriever presented with a 10‐day history of tetraplegia, depression, absent postural reflexes [65]. CSF was positive for *Leishmania* spp. DNA and a 2‐cm long mass was observed adhered to C(7) and C(8) left spinal nerves on autopsy. The spinal nerves showed fibre destruction and an inflammatory infiltrate, and cervical spinal cord sections showed multifocal, diffuse granulomatous inflammation in the white matter. Perivascular infiltrates were observed in some areas of the brain, and the parenchyma showed a subtle pallor. *L. infantum* amastigotes were found in the spinal nerves, spinal cord, brain parenchyma, and CPs, and pathology defined the neurological insults as radiculoneuritis, myelitis, and mild meningoencephalitis [65]. Another single, but atypical case of neurological leishmaniasis described a dog with signs of multifocal intracranial lesions involving the vestibular system and the cerebellum, and MRI showed a granulomatous inflammation/infection [66]. The CSF had a pronounced mononuclear pleocytosis and was positive to the Pandy Test and to *L. infantum/L. chagasi b*y PCR.

More recently, Da Rosa et al. used polymerase chain reactions (PCR) to study 200 samples taken from dogs living in an endemic region for leishmaniasis in Rio Grande do Sul, Brazil with subclinical infection but without overt neurological signs [67]. Of these, 26.5% tested positive for *L. infantum* kDNA in the brains. Histopathology showed suspected distemper in one dog, lymphoplasmacytic meningitis in another, and bilateral hydrocephalus and diffuse brain oedema in a third. In ~11% of the dog brains analysed, mild inflammatory infiltration and slight vacuolation were also observed. The authors concluded that *Leishmania* infection in endemic areas should be considered as a differential diagnosis for neurological disease.

A comprehensive study of CVL was reported for 10 dogs by Giannuzzi et al., which involved low‐field MRI, computed tomography scanning, cerebrospinal fluid (CSF) analysis and histopathology [68]. This retrospective study evaluated 8884 dogs, of which only 10 completely fulfilled the criteria of showing neurological signs associated with VL. Of these, seven showed CNS dysfunction, and three showed PNS dysfunction. Neurological diagnoses of the dogs with CNS dysfunction included (1) two dogs with non‐suppurative meningoencephalitis, with neuroanatomical localisation of encephalic multifocal syndrome, characterised by diffuse infiltrate lesions in the cerebellum, brainstem and forebrain; (2) a non‐suppurative meningomyelitis, localised to C6‐T2 spinal cord segments (central cord syndrome with an infiltrative pattern); (3) a presumptive ischaemic myelopathy, with a multifocal spinal localisation; (4) a haemorrhagic infarct in the cerebral artery supply; and (5) brainstem and cranial cervical spinal cord haemorrhage, associated with inflammation of the fourth ventricle CP. *Leishmania* kDNA was found specifically in the CSF of three dogs, and others had both DNA and high levels of anti‐*Leishmania* antibodies in the blood. Finally, one dog with CNS dysfunction that had high levels of anti‐*Leishmania* antibodies had a suspicion of a left‐sided cranial cervical spinal cord lesion, but CSF analysis showed a severe neutrophilic pleocytosis, and the diagnosis was of a secondary bacterial meningomyelitis. The remaining three dogs showed involvement of the PNS, with two dogs diagnosed with polymyositis characterised by severe muscle atrophy, and the third with a lymphocytic perivascular infiltration of the nerves and muscles [68].

To our knowledge, the first literature description of meningitis associated with CVL was made in 2001, in which two dogs showed the presence of *L. infantum* parasites in the meningeal area [69]. In these animals, the meninges showed signs of chronic inflammation with lymphocyte and plasma cell infiltrates and abundant histiocytes containing *Leishmania* amastigotes, with occasional polymorphonuclear leucocytes (PMNLs) and granulomatous lesions. The most intense inflammation was found in the perivascular cutting, although it was patchy with alternating areas of tissue destruction and normal tissue. In another study done in the Aegean region of Turkey, this time of 22 VL dogs with usual and unusual clinicopathological findings, one dog had seizures, tremors, behavioural changes, and lapsed into coma; amastigotes were detected in a few macrophages of the meninges and the perivascular areas in the brains of three dogs [70]. Other parts of the CNS involved included the spinal cord, e.g. a presumptive intramedullary spinal cord mass attributable to CVL has been reported, with positive *Leishmania* DNA in the CSF and tissue biopsy samples [71]. The presence of an extradural granuloma was also observed in a dog with VL, with histology showing the presence of free amastigotes within the granuloma and macrophages infected with *Leishmania* amastigotes [72].

Paciello et al. described a breeder group of beagles with established leishmaniasis that presented with a neuromuscular disorder characterised by exercise intolerance and muscle atrophy; these animals were diagnosed with inflammatory myopathy, caused by immune‐mediated alterations associated with *Leishmania* infection [73]. In addition to skeletal muscle atrophy, masticatory muscle atrophy in CVL has been reported [74]. Seizures have also been observed in dogs naturally infected with *L. infantum* and suffering with serum hyperviscosity syndrome that induced CNS hypoxia [75].

Dogs and cats, like humans described above, can also suffer ocular abnormalities with leishmaniasis. Dogs are considered the primary reservoir for zoonotic infection, and cats as a secondary reservoir. Approximately 25% of chronic CVL cases have ocular abnormalities, with a range from ~16–80% across various studies [24], with uveitis as the most common manifestation. Other prevalent findings included blepharitis and keratoconjunctivitis [76] and retinitis [77]. In an early study of a 6‐year‐old female dog with VL, the disease was confined to the eyes and adjacent tissues and was manifested as a severe bilateral endophthalmitis and associated blepharitis. *L. donovan*i was cultured from conjunctival swab, eyelid, anterior chamber, and vitreous body [78]. More recently, *Leishmania* amastigotes have been observed in the Meibomian glands, main lacrimal gland and nictitating membrane gland of dogs with leishmaniasis [79]. The presence of *Leishmania* parasite and the associated inflammatory response probably explain the clinical ocular signs in CVL. Ocular signs were also observed in 24 out of 150 dogs naturally infected with *L. infantum*, and included 16 cases of keratoconjunctivitis (three with keratoconjunctivitis sicca), six cases of moderate uveitis and two cases of panophthalmitis [80]. A low rate of uveitis (1%) was observed in Slappendel's study [81] of 95 dogs with VL, although the authors reported that after treatment with Glucantime, five dogs developed ulcerating kerato‐conjunctivitis (2 dogs) and iridocyclitis (3 dogs before and 3 dogs during treatment), which ultimately resulted in bilateral panophthalmia [82] in 4 dogs. Uveitis was associated with the eruption of granulomatous nodules on the palpebrae in one dog [81]. In a retrospective study of leishmaniasis in dogs, 105 dogs (24.4% of all cases) had ocular or periocular leishmaniasis, and 16 dogs (15.2% of ocular cases) had only ocular lesions and no systemic signs [76]. In addition to anterior uveitis, blepharitis and keratoconjunctivitis, eyelid lesions were not unusual [76]. These included a dry dermatitis with alopecia, diffuse blepharoedema, cutaneous ulceration, and discrete nodular granuloma formation. In addition, corneal lesions that resembled nodular granulomatous episclerokeratitis were observed in dogs with keratoconjunctivitis [76]. In another study of dogs with natural infection, granular and diffuse anti‐*Leishmania* IgG deposits were found in the ciliary body, ciliary processes, sclerocorneal limbus and iris [57]. Lesions have also been observed in the ciliary processes, ciliary body, sclerocorneal limbus, iris and lacrimal duct of two dogs with VL [83]. There were thrombosed vessels in the sclerocorneal zone and iris and intense inflammation with lymphocyte infiltrates, plasmatic cells and macrophages with amastigote forms of *Leishmania* [83]. Vasculitis with dilation and thrombi were detected in both dogs, with oedema and hyalinisation [83]. In the study from Pietro et al. [84] of sheltered and owned‐client dogs naturally infected by *L. infantum*, the prevalence of ocular lesions in 45 shelter dogs with leishmaniasis was ~71%, with blepharitis as the most frequent lesion (50%), whereas anterior uveitis was observed in only ~9% of the cases. In common with Slappendel's study, keratouveitis often occurred during or after treatment with Glucantime, which could have been due to the treatment itself or to a recurrence of systemic VL [81, 84]. In the most recent study of ocular manifestations in the eyes of dogs with *L. infantum* leishmaniasis, the main findings were keratoconjunctivitis (72%; 38/53 dogs), hyperplasia of conjunctival lymphoid follicles (55%; 29/53 dogs), blepharitis (51%; 27/53 dogs), lower levels of uveitis (21%; 11/53 dogs), with ocular production of anti‐*Leishmania* IgG detected in 74% (39/53) of infected dogs [85].

Feline leishmaniasis presents primarily as a cutaneous disease, and cats infected with *Leishmania* (*infantum* principally) also can show ocular abnormalities, although reported cases in the literature are far fewer than those for dogs [86]. The first reported case, to our knowledge, of feline ocular leishmaniasis described a granulomatous (pseudotumoral) iridociclitis [87]. In the case of an 8‐year‐old, spayed female domestic short‐haired cat, ocular histology showed a granulomatous inflammation located in fibrous and vascular layers, and the presence of large numbers of intracellular *Leishmania* amastigotes in the uveal tract, cornea, sclera and retina [88]. In a study of 15 cats infected with *L. infantum*, the main histopathological finding in the eye was a diffuse granulomatous inflammation, with clinical signs of blepharitis, conjunctivitis and panophthalmitis [89]. Chronic uveitis, keratitis and granulomatous blepharitis, with granulomatous lesions on both upper eyelids and intracytoplasmatic *L. infantum* revealed by a corneal smear, have been observed in a domestic cat [90]. In a more recent Spanish study of 16 cats with leishmaniasis, ocular disease was present in 6/16 (37.5%) cats, with clinical signs across the whole group, but varying between the cats, of corneal oedema and panuveitis, melting keratitis and corneal perforation, chorioretinitis alongside exophthalmos, chemosis with proliferative conjunctivitis, the presence of palpebral nodules, and uveitis [91]. Ocular evidence of anti‐*Leishmania* IgG antibodies has recently been demonstrated in the aqueous humour of both eyes of a 6‐year‐old female, spayed European Shorthair cat with *L. infantum* infection [92], with the animal suffering with blindness, glaucoma and high‐grade uveitis, and the presence of high numbers of *Leishmania* spp. amastigotes in histiocytes.

### 2.2 Entry of *Leishmania* Parasites Into the Brain and the Inflammatory Response

**Figure 2:**
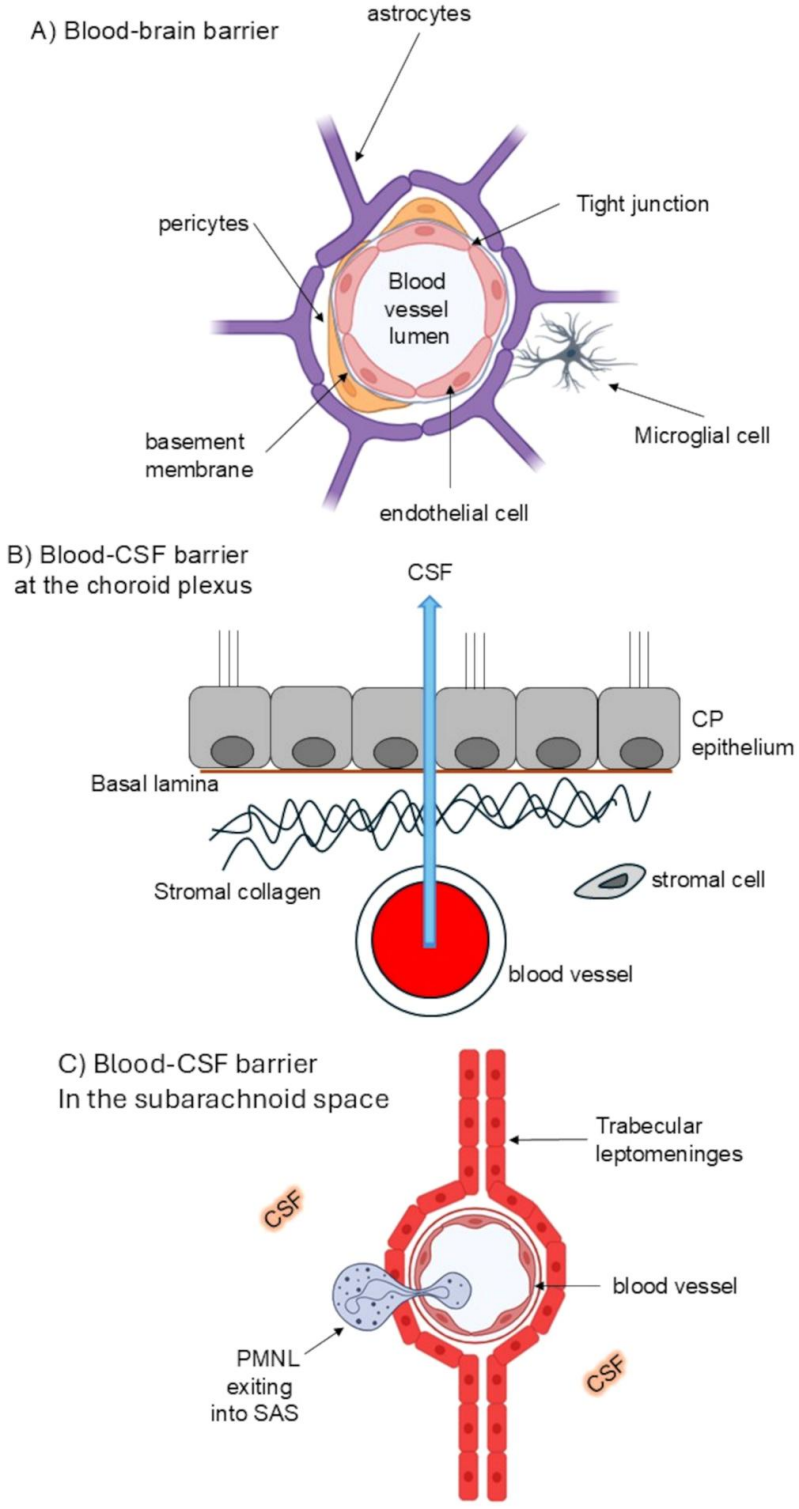
Cartoons of the (A) mammalian blood‐brain barrier (BBB); and of the blood‐cerebrospinal fluid barriers (BCSFB) at the (B) choroid plexus (CP) and (C) the subarachnoid space SAS). The anatomy of the BBB and BCSFB are significantly different, although both do not appear to present barriers to *Leishmania* invasion.

In mammals, the CNS has four major anatomical fluid compartments—blood, CSF, interstitial (extracellular) fluid and intracellular fluid. In humans, the blood‐brain parenchyma barrier (BBB) consists of blood vessel endothelial cells with tight junctions, surrounded by a basement membrane and invested astrocyte processes and pericytes [93] and the canine BBB is structurally and functionally similar [94, 95] (Figure 2A). In humans and dogs, the blood‐CSF barrier (BCSFB) is located at two sites. It exists between plasma and the CSF at the CP (Figure 2B) and the stromal arachnoid cells (derived from the leptomeninges) produce collagen bands, and the ventricular face of the CP consists of an epithelium of large cuboidal cells derived from the ependyma. The site of the BCSFB is located where the epithelial cells are joined by these tight and gap junctions; thus, blood components can pass into the stroma but not into the CSF [93]. The other BCSFB is in the subarachnoid space (SAS) that can be penetrated by pathogens, but the process is unclear. Anatomically, there are differences between venules in the brain and those within the SAS, and this can be demonstrated experimentally by intracerebral injection of cytokines (albeit in the mouse), whereby PMNLs do not enter the brain, but there is a large infiltration of inflammatory cells into the leptomeninges, CP and ventricles [96]. Thus, PMNLs can migrate readily from leptomeningeal veins/venules into the CSF of the SAS and this route could be used by pathogens. However, the mechanism remains unclear, and pathogens would need to breach the thin‐walled vessel endothelium, the basal lamina and the surrounding leptomeningeal cell layer to gain access to the CSF (Figure 2C) [93]. A ‘Trojan Horse’ mechanism for entry is also possible, demonstrated by many microbial pathogens via intracellular hijacking of myeloid cells [97, 98, 99, 100, 101].

Hypotheses can be generated on the mechanisms of *Leishmania* entry into the CSF and brain, supported by several observational studies. To our knowledge of the literature, the first detection of *L. infantum* kDNA in the brains of naturally infected dogs was made by Grano et al., wherein *L. infantum* kDNA was detected in the frontal lobe, hippocampus, diencephalon, temporal lobe, occipital lobe, and midbrain, brainstem and CP [102]. In a subsequent study of 24 mongrel adult dogs (22 with clinical infection, 2 with neurological signs, and 2 with subclinical infection) aged between 2 and 5 years and naturally infected by VL, *L. infantum* kDNA was detected in 23/24 of the infected dogs [103]. The kDNA was found in the meninges, frontal cortex, thalamus, cerebellum, and CP of the lateral ventricles and fourth ventricle, demonstrating that the parasite could have entered the brain parenchyma. Notably, several of these dogs were co‐infected with either *Ehrlichia canis, Babesia vogeli* and/or *Toxoplasma gondii*; however, a note of caution in interpretation is that the presence of DNA detected by PCR does not prove the presence of the pathogen within the tissues. For the whole parasite, a CVL case of PNS and CNS infection caused by *L. infantum* did have amastigotes present in the spinal nerves and cord, the brain parenchyma, and within CPs [65]. *L. infantum* amastigotes have also been detected in the canine CP [58]. Thus, the presence of amastigotes inside brain blood vessels and CSF suggests disruption of the BBB and BCSFB and direct penetration of *Leishmania* to the parenchyma and meninges. *Leishmania* invasion of the CNS was further confirmed in a histopathological of 14 dogs with VL, who exhibited a mononuclear inflammatory reaction, haemorrhage, chromatolysis and gliosis [104]. In this study, *L. infantum* amastigotes were identified in the telencephalon, hippocampus, thalamus and caudal colliculus of 8 dogs, and parasite DNA was detected by PCR in the telencephalon, thalamus, hippocampus, cerebellum, caudal and rostral colliculus [104], but, surprisingly, neurological manifestations were absent. In addition, a study with an *in vitro* Schwann cell line suggested that these essential glial cells of the PNS could be reservoirs for *L. amazonensis* [105].

Several lines of evidence suggest dysfunction of the BCSFB at the CP during canine leishmaniasis, gathered from studies of samples taken from 19 dogs naturally infected with *Leishmania* spp [106]. Mild‐to‐severe inflammatory infiltrates were found mainly in the meninges and CP, and parasite DNA in the CP of the dogs. In a study of 42 mixed‐breed adult dogs in an area in Brazil endemic for VL [107], histology of brain sections showed an intense inflammatory infiltrate of CD3^+^ T lymphocytes in the CPs and brains. Sakamoto et al also described CD3^+^ T lymphocytes in several encephalic regions of dogs with *L. chagasi* VL [108]. Thus, the CP appears to be important for T cell infiltration, and the presence of anti‐*Leishmania* antibodies and albumin in the CSF further indicates that the BCSFB and the BBB were compromised. Notably, RANTES (CCL‐5) chemokine gene expression was increased in the CP of infected dogs, compared to uninfected controls, and there was a tendency for gene expression of interferon gamma‐induced protein 10 (IP‐10, CXCL‐10) [106]. Indeed, pro‐inflammatory cytokines predominate in the brains of dogs naturally infected with *L. infantum*, with upregulation of IL‐1β, IFN‐γ, and TNF‐α levels and downregulation of IL‐10, TGF‐β, and IL‐12p40 [109]. However, these expression levels did not correlate with parasite load, suggesting that CNS alterations were due to canine immune responses, irrespective of the CVL stage. Pathologically, these infected dogs showed mild to severe perivascular mononuclear cell infiltrates in the leptomeninges, CP, and periventricular areas, minimal to moderate perivascular mononuclear cell infiltration in the brain parenchyma, satellitosis and neuronophagia, and focal and diffuse gliosis [109].

Mechanistically, *Leishmania* infection with a pro‐inflammatory profile can alter BBB/BCSFB permeability and lead to leucocyte infiltration. Host cytokine and matrix metalloproteinase (MMP) activity both contribute to barrier dysfunction. MMPs are proteolytic enzymes that are implicated in BBB/BCSFB disruption and inflammation in the CNS. Brain fragments of nervous tissue from oligosymptomatic (*n* = 9), symptomatic (*n* = 8), neurological (*n* = 12) and normal dogs (*n* = 8) were examined for MMP‐2 and MMP‐9 involvement, and revealed latent form MMP‐2 in inflammatory cells inside and outside blood vessels and latent form MMP‐9 in endothelial cells and in the ependyma [110]. Both enzymes were also studied during natural systemic infection of dogs with *L. chagasi* [111]. Whereas latent and active forms of MMP‐2 were detected in some infected and control dogs, and especially high levels in the latter, latent and active forms of MMP‐9 were elevated only in some dogs with VL. By contrast, the study from Melo et al. demonstrated the large presence of active MMP‐2 in the CSF of dogs with VL [112]. Mechanistically, MMPs cleave type IV collagen, the main component of the basal membrane, and therefore contribute to barrier dysfunction and the ingress of inflammatory cells [113]. Furthermore, it is possible that extracellular release of virulence factors such as the *Leishmania* zinc‐dependent metalloprotease (GP63, leishmanolysin) is involved in the proteolytic process [114].

Expression of a plethora of cytokines is also key to breaking down the BBB/BCSFB. However, Toll‐*like* Receptor (TLR) expression does not appear to play a significant role in the brains of dogs with VL [115]. A detailed discussion of the cytokines involved in vessel endothelial cell activation, inflammation, and PMNL ingress into the brain is outside the scope of this review and can be sought elsewhere [116]. The histological alterations in the CNS of dogs with VL may be due to the up‐regulated action of pro‐inflammatory cytokines, and the absence of neuroprotective cytokines such as IL‐10, TGF‐β, and IL‐12p40 may also contribute to brain lesions [109]. Analysis of brain tissue of dogs infected with *L. chagasi* indicated an intense histological labelling of astrocytes or microglia, and these cell types could be involved in the inflammatory response and potentially the pathogenesis of neurological disorders observed with CVL [117]. In these dogs, glial reactivity correlated with T‐lymphocyte infiltration and CSF anti‐*Leishmania* antibody titres and the possibility of seizures [109].

**Figure 3:**
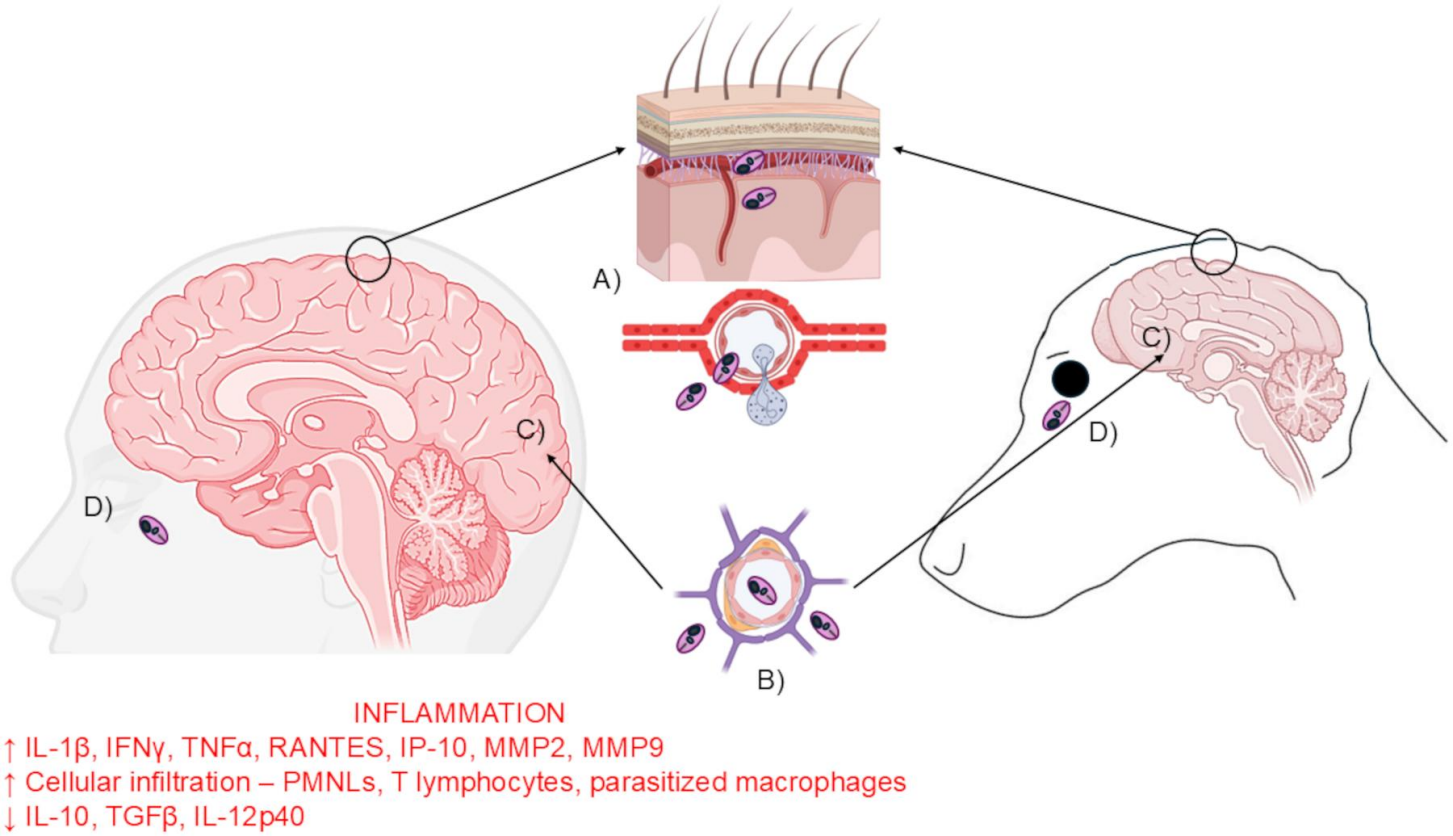
Cartoon summarising cerebral and meningeal leishmaniasis. (A) Invasion of the BCSFB by *Leishmania* for parasite entry into the meninges, including via a possible ‘Trojan Horse’ mechanism. (B) Penetration of the BBB by *Leishmania* for parasite entry into (C) cortical matter (D) Ocular involvement with parasite found in ocular structures. The outcome of infection is an intense inflammatory response, with cellular infiltration, and accompanying pathological changes, which are described in detail in the text Created in part with https://BioRender.com.

One other suggestion for parasite entry into the brain and meninges is a ‘Trojan horse’ mechanism via myeloid cells. There is direct experimental evidence that leucocytes infected with other eukaryotic pathogens, e.g., *Cryptococcus* and *Toxoplasma*, can cross the BBB [118, 119], and this is biologically plausible for *Leishmania*, although direct *in vivo* proof is lacking. For example, *L. major* can survive in PMNL granulocytes and use them as ‘carriers’ that then undergo apoptosis and are ingested by macrophages [120]. Viscerotropic *Leishmania* species that produce a bloodstream parasitaemia are more likely to reach the CNS than strictly cutaneous *Leishmania* species, and the mechanisms are probably multiple, and involve altered BBB/BCSFB permeability from inflammation, possibly direct endothelial infection/transcytosis (Figures 2, 3) and a hypothetical ‘Trojan horse’ mechanism via infected leucocytes/macrophages.

### 2.3 The Contribution of Animal Models for Studying Cerebral Leishmaniasis

As described above, the pathogenesis of leishmaniasis can be studied during natural infection and laboratory‐controlled infections *in vivo* and *ex vivo*. Pathogenesis involves the interplay of components of the parasite and the host immune response, and several mammalian models have been described for studying the outcomes of *Leishmania* infection. Rodents are commonly used to study *Leishmania* infection, including brain involvement and the inflammatory response. They are also useful for testing experimental vaccines and drug treatments [121, 122]. Loeuillet et al. has reviewed the mouse as an experimental model for leishmaniasis [123], but the involvement of the brain is our specific and more detailed focus. When BALB/c mice were infected with high inocula of *L. chagasi* (10^7^–10^8^ parasites/mouse), they developed an exponential parasite burden within internal organs such as the bone marrow and brain, and more so in the spleen and draining lymph nodes [124]. In an experimental model of TL, subcutaneous infection of BALB/c and Swiss mice with 10^4^ *L. amazonensis* amastigotes resulted in a discrete hyperaemia and infiltration of mononuclear cells and neutrophils into the meninges, but not parasites (which may be solely inoculum dependent) [125]. Necrosis and the presence of parasitised macrophages were observed in the cerebral parenchyma, and CD8^+^ T lymphocytes were the major cell type in the CNS inflammatory infiltrate, with smaller numbers of CD4^+^, CD11b, and dendritic cells [125].

Brain infection and inflammation were studied in‐depth in the BALB/c mouse model of visceral and cerebral leishmaniasis using bio‐luminescent *L. donovani* and real‐time 2‐ and 3D imaging tools [126]. In this study, parasites were detected within the cortex and base of the brain as early as 3‐days post infection. Infection induced a compartmentalised inflammatory response in the cortex and brain base (higher in the cortex), which consisted of an early phase (days 3–14) that was characterised by (i) the influx of neutrophils and Ly6C^high^ macrophages, and the presence of (ii) chemokines and their receptors (CCL‐7, CCL‐5, CCL‐12, CXCL‐10), (iii) pro‐inflammatory cytokines (IL‐1β, IL‐6, IFN‐ γ) and (iv) anti‐inflammatory cytokines (IL‐10, TGF‐β). A ‘re‐inflammation’ phase occurred 90 days later with only pro‐inflammatory cytokines (IL‐1β, IL‐6, IFN‐ γ) and MMP‐9 up‐regulated [126]. Although this BALB/c mouse has provided useful information on the inflammatory processes occurring in the brain infected with *Leishmania* parasites, there is a notable caveat ‐ namely that mice were injected with a dose of 5 × 10^7^ bio‐luminescent *L. donovani* promastigotes in 150 μL of PBS by the intraperitoneal route, and thus the model is artificial in nature [126].

BALC/c mice have also been infected with *L. amazonensis* via footpad injection, and in this model, infected mice showed a decrease in IL‐1, IL‐4, IL‐6, IL‐10 and IFN‐γ cytokines, brain‐derived neurotrophic factor and nerve growth factor in the prefrontal cortex, at 60‐ and 120‐days post‐infection [127]. Parasite kDNA was found in the brains at 120 days and the levels of TNF‐α and IBA‐1 (ionised calcium binding adaptor molecule 1, a marker of microglia) were increased by 120 days also. The inflammatory response was accompanied by increased anxiety behaviours, and the normalisation of cytokine and neurotrophic factors was accompanied by a normalisation of behaviour [127]. By 6 months, *L. amazonensis* infected mice had decreased levels of TNF‐α and IL‐6 in the prefrontal cortex (compared to 4 months), which suggested regulation of microglia activation.

In addition to cytokines and chemokines, biochemical markers of neurodegenerative diseases with a pro‐inflammatory profile are also manifest during VL. A subcutaneous footpad infection of BALB/c mice with *L. amazonensis* resulted in an increase in tau phosphorylation (Ser(396)) and up‐regulation of the Receptor For Advanced Glycation Endproducts (RAGE) in the brain cortex [128]. Infected mice showed increased protein carbonylation, decreased IFN‐γ levels and diminished antioxidant defences in brain tissue, but the levels of TNF‐α, IL‐1β, and IL‐6 were like uninfected mice. Thus, neurological symptoms in chronic leishmaniasis were associated with the disruption in CNS protein homoeostasis that resulted from oxidative stress. Indeed, the administration of antioxidant treatment to infected mice inhibited tau phosphorylation and recovered IFN‐γ to control levels [128]. In another study, untargeted metabolomics using gas chromatography and mass spectrometry was used to examine the metabolome within BALB/c mouse tissues and biofluids after infection with *L. donovani* [129]. Metabolites unique to the brain were identified as well as potential biomarkers, e.g. arginine, fumaric acid, oxalosuccininc acid and phosphopyruvic acid, which were up‐regulated during infection. However, like the artificial BALB/c mouse model of *L. donovani* infection described above, the BALB/c mouse infected with *L. amazonensis* via footpad inoculation [127, 128] is not an appropriate model to study possible brain involvement in CL. It is likely that brain involvement in this rodent is the result of uncontrolled cutaneous lesions and disseminating infections that do not reflect CL in humans or dogs.

A model of experimental ocular leishmaniasis caused by *L. amazonensis* has been developed in C57BL/10 and BALB/c mice [130]. Mice inoculated by the intravitreal route developed severe lesions with parasites observed in the anterior region of the eye at 60 days after infection. The C57BL/10 mice had cells containing parasitophorous vacuoles associated with pigmented cells and an inflammatory infiltrate that included mast cells. The mouse models were similar for parasite infection and clearance and appeared to mimic the ocular lesions described in symptomatic dogs in endemic areas of VL.

Golden hamsters (*Mesocricetus auratus*) are also a valuable model for research into *Leishmania* vaccines, drugs and the pathogenesis of infection [121, 131, 132]. However, little has been communicated on brain involvement during infection, with only a report of hamsters infected with bone marrow from humans with VL showing parasite invasion of the spleen, liver, kidneys, lungs and heart, and notably most of the animals (> 80%) with parasitism within the brain [133]. Thus, the evidence of CNS involvement in rodent models of infection appears robust. By contrast, to our knowledge, there are currently no published studies demonstrating the presence of the parasites in the CNS of non‐human primates, even though they can be naturally and experimentally infected with *Leishmania* spp. Most reports have focused on systemic visceral infection, clinical disease, and vector transmission [134, 135, 136].

As explained above, the dog is the primary zoonotic host for natural infection with *Leishmania infantum*, and most studies of brain involvement have been done in naturally infected dogs. However, there are several studies that describe experimental laboratory infection of dogs, e.g. to establish long‐term cellular immune reactions to *Leishmania* antigens [137], a cutaneous *Leishmania* infection model [138], and experimental infection models for *L. infantum* [139, 140, 141] and visceral *L. donovani* [142]. In addition, dogs were exposed to *L. infantum* along with sand fly saliva, to mimic a more natural transmission [143]. This latter model demonstrated the immunomodulatory role of saliva and has been used to evaluate salivary protein–based vaccines, supporting a more comprehensive model design for real‐world applicability.

An interesting animal model that juxtaposed *Leishmania* and a human neurological condition unrelated to leishmaniasis, was a Wistar rat model of Alzheimer's disease, in which the disease was induced with amyloid‐β peptide, following which the rats were treated with crude antigen preparations from *T. gondii, L. major* and hydatid cyst [144]. The application of *L. major* antigens appeared to have neuroprotective effects that reduced oxidative stress, apoptosis, chemokine production and the histopathological changes that occurred, especially in the hippocampi, of Alzheimer's disease rat brains. This potential neuroprotective effect was also seen with an in vitro model of peripheral blood mononuclear cells (PBMC), previously primed with lipopolysaccharide (LPS) and stimulated with nigericin, to test the hypothesis that parasite infection could reduce the NRLP3 inflammasome, which plays a major role in the inflammation observed with Alzheimer's disease [145]. Infection of this model with *L. infantum* significantly reduced ASC‐speck formation (i.e. intracellular inflammasome proteins assembly) and the production of activated caspase 5 and IL‐1β, but increased production of activated caspase 1 and IL‐18. Moreover, infection also suppressed the production of TNF‐α and increased IL‐10. Thus, *Leishmania* antigens may have anti‐inflammatory effects that hamper neuroinflammation in leishmaniasis and favour intracellular parasite survival.

Complementary to *in vivo* models, cell cultures maintained *in vivo* can be useful for studying aspects of *Leishmania* pathogenesis. Infection studies on an *in vitro* co‐culture model containing murine bone marrow‐derived macrophages and astrocytes supported the hypothesis that astrocytes could lead to a reduction of *Leishmania* parasites in the CNS [146], accompanied by increased IFN‐γ and IL‐6 in the co‐cultures. *Ex vivo* primary neonatal rat glial cells have been used as a model to evaluate new drug treatments, e.g. Glucantime treatment of cultures infected with antimony‐resistant *L. tropica* amastigotes reduced parasite load [147, 148].

## 3 Conclusions and Limitations

Leishmaniasis is a global NTD that continues to afflict humans and other mammals, including dogs. The classical clinical signs of leishmaniasis in humans range from self‐healing cutaneous lesions, disfiguring mucosal disease to fatal visceral infection. Dogs can suffer from chronic and systemic infections that can also be fatal. The evidence compiled in this review shows that the clinical and neurological symptoms in mammals infected with *Leishmania* spp., coupled with histopathological findings and immunological studies, are demonstrative of direct interactions between the parasite and the CNS and PNS (Figure 3). However, some limitations need to be acknowledged. Most available data on the occurrence of neurological manifestations and CNS involvement in leishmaniasis come from case reports, small clinical cohorts, and naturally infected dogs, which limits the generality of findings to human disease. Demonstration of parasite DNA in the brain or CSF, while valuable, does not confirm the presence of viable parasites or active infection, and histopathological confirmation remains scarce. Mechanistic insights into how *Leishmania* crosses the BBB and BCSFB barriers are largely speculative, with hypotheses such as endothelial disruption or “Trojan horse” mechanisms yet to be validated *in vivo*. The relationship between neuropathological changes and clinical neurological symptoms remains poorly defined, and there is a lack of longitudinal or quantitative studies that correlate parasite burden, the host immune response, and disease outcomes. Experimental models are dominated by a limited range of *Leishmania* species and rodent hosts, which may not fully mimic human or canine neuro‐infection. Future research should focus on establishing standardised experimental and diagnostic criteria for neuro‐leishmaniasis, and integrate molecular, immunological, and imaging approaches to elucidate the mechanisms of CNS invasion, persistence, and pathology. Comparative studies across *Leishmania* species and host systems, coupled with translational research to identify diagnostic biomarkers and therapeutic drug and vaccine targets, will be essential to advance our understanding of this underappreciated aspect of leishmaniasis.

## Conflicts of Interest

The authors declare no conflicts of interest.

## Supporting information


Supporting File

## Data Availability

The authors have nothing to report.
