# Supplementary material for: Brain Involvement in Leishmaniasis
Source: Cell Biochem Funct. 2026 Apr 11;44(4):e70209. doi: 10.1002/cbf.70209 (PMC13069917; doi:10.1002/cbf.70209)
Supplement: Supplementary file 1 — Supporting File [file CBF-44-e70209-s001.docx]

**Glossary of terms**

| **Term** | **Meaning** |
| --- | --- |
| Aniscoria | Unequal pupil sizes |
| Bilateral mydriasis | Abnormal dilation of both pupils |
| Bilateral panophthalmia | Severe condition that affects the bilateral eye structures or tissues, leading to inflammation and potential blindness. |
| Blepharitis | Inflammation of the eyelids |
| Blepharoedema | Edema of the eyelids, causing swelling |
| Choroiditis | Inflammation of the choroid of the eye |
| Choroiditis | Inflammation of the choroid of the eye |
| Chromatolysis | Disintegration or dispersal of the basophilic nissl bodies, neuronal cell body |
| Dysphagia | Difficulty in swallowing |
| Endophthalmitis | Inflammation of intraocular vitreous and aqueous fluids |
| Episclerokeratitis | Unique to dogs; inflammation of the episclera, the tissue between the conjunctiva and the sclera of the eye. It can manifest as either nodular or diffuse forms. |
| Epistaxis | Nosebleed |
| Exophthalmos | Protrusion of eyeball from eye socket |
| Facial ptosis | ‘droopy’ eyelid |
| Granulomatous iridocyclitis | Inflammation of the iris and the ciliary body |
| Haemophagocytic lymphohistiocytosis (HLH) | Overactivity of immune cells leading to excessive inflammation |
| Hyperemia | Excess of blood to organs |
| Ischaemic myelopathy | Blood supply to part of the spinal cord suddenly interrupted by a clot that blocks a small artery |
| Leukocytoclastic vasculitis | Small blood vessel inflammation |
| Lymphoplasmacytic | Inflammation involving lymphocytes and plasma cells |
| Masticatory muscle atrophy | Decrease in muscle mass and function of the muscles involved in  chewing |
| Myoclonus | Uncontrollable movement that includes sudden, brief involuntary twitching, jerking, or spasm of a single muscle or a group of muscles |
| Neuronophagia | Dying neurons surrounded by microglial cells |
| Non-suppurative meningoencephalitis | Inflammation of the meninges and brain without pus |
| Onychogryphosis | Thickening and curvature of the nails. |
| Panophthalmitis | Inflammation of all layers of the eye |
| Paraparesis | Partial paralysis of limbs |
| Polymyositis | Chronic muscle inflammation and weakness |
| Satellitosis | Abnormal clustering of one cell type around another |
| Serum hyperviscosity syndrome | Thickened blood with overall decrease in blood flow |
| Strabismus | Misalingment of the eyes (squint) |
| Tetraparesis | Quadriparesis, i.e. four limbs suffer muscle weakness |
| Tetraplegia | Quadriplegia, i.e. paralysis of all four limbs |

**Glossary of abbreviations**

| **Abbreviation** | **Meaning** |
| --- | --- |
| NTD | Neglected Tropical Disease |
| TL | Tegumentary Leishmaniasis |
| VL | Visceral Leishmaniasis |
| CL | Cutaneous Leishmaniasis |
| ML | Mucosal Leishmaniasis |
| CDL | Cutaneous-diffuse Leishmaniasis |
| IFNɣ | Interferonγ |
| IL | Interleukin |
| TNF-α | Tumour Necrosis Factor-α |
| TGF-β | Transforming growth factor-β |
| YY1 | Yin Yang 1 |
| CNS | Central Nervous System |
| PNS | Peripheral Nervous System |
| L-AMB | Liposomal amphotericin B |
| Sb5+ | Sodium stibogluconate |
| CVL | Canine Visceral Leishmaniasis |
| CP | Choroid Plexus |
| PMNL | Polymorphonuclear Leucocytes |
| BBB | Blood Brain Barrier |
| CSF | Cerebrospinal Fluid |
| BCSFB | Blood Cerebrospinal Fluid Barrier |
| SAS | Subarachnoid Space |
| MMP | Matrix Metalloproteinase |
| TLR | Toll Like Receptor |
| CCL-5, -7, -12 | Chemokine (C-C motif) ligand |
| CXCL-10 | C-X-C motif chemokine ligand |
| IBA-1 | Ionized calcium binding adaptor molecule 1 |
| RAGE | Receptor For Advanced Glycation Endproducts |
| PBMC | Peripheral Blood Mononuclear Cells |
| NRLP3 | NLR (nucleotide-binding oligomerization domain-like receptors) family pyrin domain containing 3 |
| LPS | Lipopolysaccharide |
| HIV | Human Immunodeficiency Virus |
| FLAIR | FLuid-Attenuated Inversion Recovery |
| HLH | Haemophagocytic Lymphohistiocytosis |
| kDNA | Kinetoplast DeoxyriboNucleic Acid |
